# Myopericytoma arising from myopericytosis—a hitherto unrecognized entity within the lung

**DOI:** 10.1007/s00428-020-02972-9

**Published:** 2020-11-26

**Authors:** Ulrike Gruber-Moesenbacher, Alicia Morresi- Hauff, Katja Behr, Helmut Popper

**Affiliations:** 1 Institute of Pathology, Helios Clinics, Gauting, Germany; 2 Institute of Pathology, Mittelthueringen, Bad Berka, Germany; 3 Institute of Pathology, Medical University of Graz, Neue Stiftingtalstrasse 6, 8036 Graz, Austria

**Keywords:** Myopericytosis, Myopericytoma, Pericytic tumor, Lung

## Abstract

Two cases of myopericytosis combined with pericytoma originating within the lung are reported. These are rare pulmonary tumors. The differential diagnosis for hemangiopericytoma and pericytic tumors with glomus elements is discussed. Both myopericytic lesions mimic other lesions, which are more commonly seen in the lung. Based on the expression of vascular growth factor receptors 2 and 3, an antiangiogenic therapy was suggested for the patient with the myopericytoma. A treatment with an angiogenesis inhibitor resulted in a regression of the tumor, but not the precursor lesion. Probably a more specific therapy using tyrosine kinase inhibitors for VEGFR2/3 might better control these myopericytic proliferations.

## Introduction

Pericytic tumors are rare tumors encountered in the lung. They comprise a spectrum of hemangiopericytoma, infantile myofibromatosis, tumors with glomus elements, and smooth muscle cells. They all show a perivascular proliferation intimately associated with blood vessels. They can form large tumors but also diffuse spreading lesions with multicentricity. The vascular structures can be prominent as in hemangiopericytoma, or branching vessels with epithelioid cells as in glomangiopericytoma. Some tumors lack these prominent vascular structures but show more smooth muscle cell proliferations as in myopericytoma [8]. Most tumors in this pericytic lineage have been described in soft tissues and skin, whereas they are rare in the lung [1, 20, 33]. Another tumor, solitary fibrous tumor, which is frequently encountered in the pleura may also occur within the lung and previously was confused with pericytic tumors. However, due to its characteristic molecular alteration (STAT6-NAB2 fusion), SFT can be clearly separated from the pericytic tumors [26]. Here, we report on two precursor lesions for myopericytoma arising primarily within the lung with quite different morphologies, in one case also with a subsequent tumor development. In addition, we identified a treatment option for this kind of tumors.

## Clinical history

### Case 1

A female patient aged 41 years presented with bilateral reticulonodular infiltrates. An open lung biopsy was taken. Due to some small cysts and the bilateral infiltration, a different diagnosis was made in an outside pathology department and by a consultant.

**Fig. 1:**
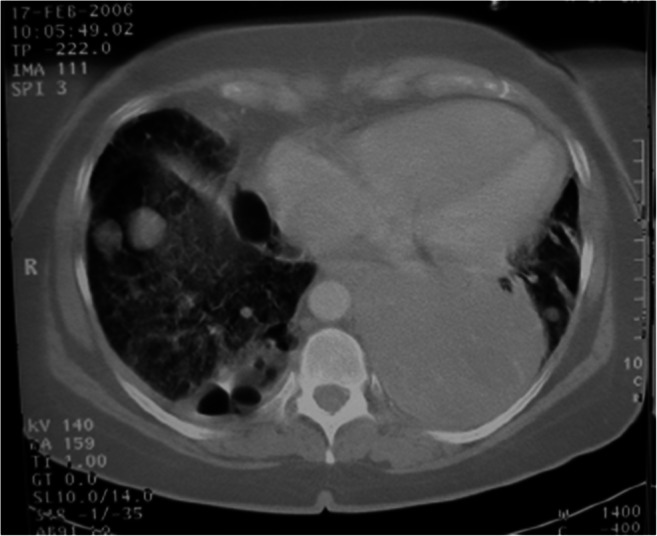
CT scan showing the large tumor in the left lung of patient 1

Five years later, the patient presented with the same reticulonodular infiltrations but in addition with a large nodular mass in the left hemithorax (Fig. 1). A fine needle biopsy was taken from the mass. This biopsy was received for consultation and also the original tissue was submitted for re-review.

After a diagnosis was established, the patient initially was treated with antihormonal therapy because of focal positivity of tumor cells for progesterone receptor. Surgery was excluded because of the preexisting diffuse process in both lungs. As this did not result in any improvement, chemotherapy was installed but withdrawn because of tumor progression. Based on the results of the pathological examinations and suggestions for a change in the therapy protocol, gemcitabine combined with bevacizumab was administered. This resulted in a dramatic tumor shrinkage within 6 weeks, but the reticulonodular infiltrates did not change much. During the antiangiogenic therapy, massive bleeding occurred, which necessitated interruption of the treatment. After 2 months, a recurrence of the tumor was noted on control CT scan, and the patient was asked to continue the therapy. However, the patient refused further treatment and finally died 2 months later. No autopsy was performed.

### Case 2

An 82-year-old female patient presented with a tumor nodule in the left lower lobe, and in addition with small trabecular infiltrations. In addition to a tumor, also lymphangioleiomyomatosis was suspected. An open lung biopsy was taken. An adenocarcinoma was diagnosed but additional nodular and trabecular infiltrations were recognized, which did not fit into any suspected diagnosis. Therefore, the tissues were sent for consultation.

## Material and methods

Tissue blocks were received from both patients. Four-micrometer-thin sections were stained by hematoxylin and eosin (H&E), followed by a series of immunohistological investigations (Table 1). An informed consent was received from the patients; an ethical vote was issued by the Ethics Committee of the Medical University (EK 24-135 ex 11/12). All clinical data were anonymized.

**Table 1:** List of antibodies, dilution, and visualization

Antibody	Source	Dilution	Visualization
Pancytokeratin	Dako clone HNF116,	1:100	Ventana ultraView
CD 56 (NCAM)	Novocastra clone 1B6	1:100	Ventana ultraView
LCA CD45	Dako clone PD7+2B11	1:4000	
S100 protein	Dako	1:2000	Protease XXIV Dako ENV (K5007)
Vimentin	Linaris clone V9	Ready to use	
Desmin	Dako clone DE-R11	1:200	
Smooth muscle actin	Sigma clone 1A4	1:5000	CC1 Ventana ultraView
CD31	Dako clone JC70A	1:50	Protease XXIV Dako ENV (K5007)
CD34	Neomarkers clone QBEnd10	1:800	CC1 Ventana ultraView
CD68	Dako clone KP1	1:300	Prot1 Ventana iview
Ki67	Ventana clone K2	Ready to use	
HMB45	Dako clone HMB45	1:100	
ER	Dako clone 1D5	Ready to use	
PG	Dako clone PgR636	Ready to use	
VEGF-A	Santa Cruz clone 1-20	1:500	MW Tris Dako ENV (K5007)
VEGFR-2	Santa Cruz clone A3	1:50	CC1 Ventana iview
VEGFR-3	Santa Cruz clone c-20	1:200	CC1 Ventana iview
Endothel kinase Tie2	Santa Cruz clone H176	1:50	CC1 Ventana iview
cAMP kinase alpha	Santa Cruz	1:200	
Hamartin	Santa Cruz clone H300	1:50	CC1 Ventana iview
Tuberin	Santa Cruz clone N19	1:100	CC1 Ventana iview
Calretinin (SP65)	Ventana 790-4467	Rtu	CC1 Ventana ultraView
Podoplanin D2-40	Dako M3619	1:100	MW6,0 Dako ENV(K5007)
Factor VIIIAG (F8/86)	Dako M0616	1:1000	Prot1 Ventana iview
HHV8	Novocastra 13B10	1:25	
CD35	Dako clone Ber MAC DRC	1:10	
Cytokeratin 7 Clone OV-TL	Dako M7018	1:100	Protease Dako ENV (K5007)
CAM5.2	BD Bioscience 345779	Rtu	Prot1 Ventana ultraView
AE1/3	Dako M3515	1:50	Protease Dako ENV (K5007)
Chromogranin A Ab3	Thermo Scientific MS-382-P	1:3000	CC1 Ventana ultraView
Synaptophysin	Dako M7315	1:50	CC1 Ventana ultraView
Estrogen receptor EP1	Dako IR084	Rtu	Dako Omnis high pH
Progesterone receptor PgR1294	Dako GA090	Rtu	Dako Omnis high pH
TTF1 (8G7G3/1)	Dako 3575	1:100	MW9,0 Dako ENV (K5007)
P40 BC28	Ventana 790-4950	Rtu	CC1 Ventana OptiView
HMB45	Dako GA052	Rtu	Dako Omnis high pH
MelanA A103	Dako IR633	Rtu	Dako Omnis high pH
MITF Clone D5	Dako M3621	1:40	MW Tris Dako ENV (K5007)
MIB1 Ki67	Dako GA6266	Rtu	Dako Omnis low pH
Vimentin C9	Dako GA630	Rtu	Dako Omnis high pH
Desmin D33	Cell Marque 243M-16	1:50	CC1 Ventana ultraView
Factor XIII	Calbiochem 233498	1:1000	Prot1 Ventana iview
ERG EPR3864	Abcam ab133264	1:200	CC1 Ventana ultraView
S100 protein	Dako GA504	Rtu	Dako Omnis low pH
Caldesmon h-CD	Dako GA054	Rtu	Dako Omnis high pH
MyoD1 5.8A	Dako M3512	1:50	MWDako6,0 Dako ENV (K5007)
Cathepsin K	Abcam ab37259	1:1000	CC1 Ventana ultraView
PDGFR beta	Neomarkers RB1692-P	1:100	MW pH 9.0, 150W, Dako Envision K5007 DAB

## Results

### Morphology of diffuse and nodular lesions of case 1

**Fig. 2:**
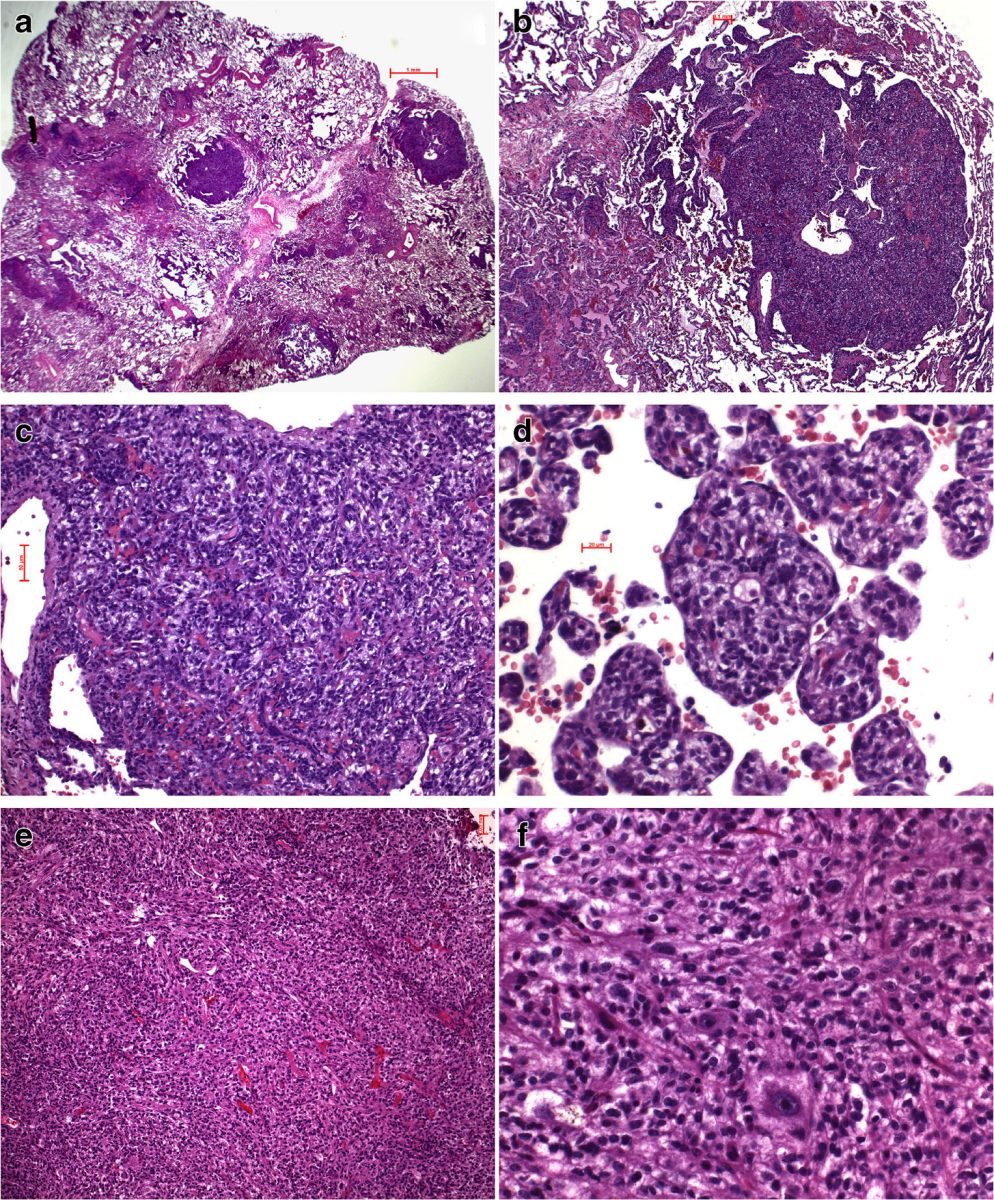
Case 1: **a** Overview with two nodules and diffuse dark-stained precursor lesion. H&E, bar 1 mm; **b** and **c** medium and high magnification of the proliferation in the nodules. Note the spindle cells with small clear cytoplasm and their intimate association with blood vessels. H&E, bars 0.1 mm and 50 μm. **d** High magnification of the diffuse lesion. The lesion is composed of spindle cells with small clear cytoplasm closely associated with small blood vessels. H&E, bar 20 μm. **e** Overview of the myopericytoma. The tumor cells are associated with thin-walled blood vessels and are spindle shaped. H&E, bar 0.5 mm. **f** High magnification of the tumor cells. There are more epithelioid clear cells, some of them positive for HMB45, some giant cells with large nuclei, and also cells with myofilaments. H&E, × 400

Four years before the tumor was recognized, open lung biopsies had been taken, because of diffuse bilateral lung infiltrations; a Langerhans cell histiocytosis was suspected. In these biopsies, several nodular lesions with a diameter of up to 1.2 mm were seen in addition to diffuse dark-stained infiltrations (Fig. 2a). On higher magnification, a dense infiltration by cells with round-to-spindle-shaped dark nuclei and a basophilic, focally clear cytoplasm was seen in the nodular and diffuse lesions (Fig. 2b, c). A few larger cells with clear cytoplasm were seen (Fig. 2d). These cells showed an intimate association with blood vessels. The pneumocyte layers were unaffected.

The tumor was composed of similar cells (Fig. 2e), but there were more clear cells and even some giant cells (Fig. 2f). Focally, cells presenting with spindle cell morphology and cytoplasmic filaments were suggestive for smooth muscle cells (Fig. 2f).

### Morphology of trabecular lesions in case 2

**Fig. 3:**
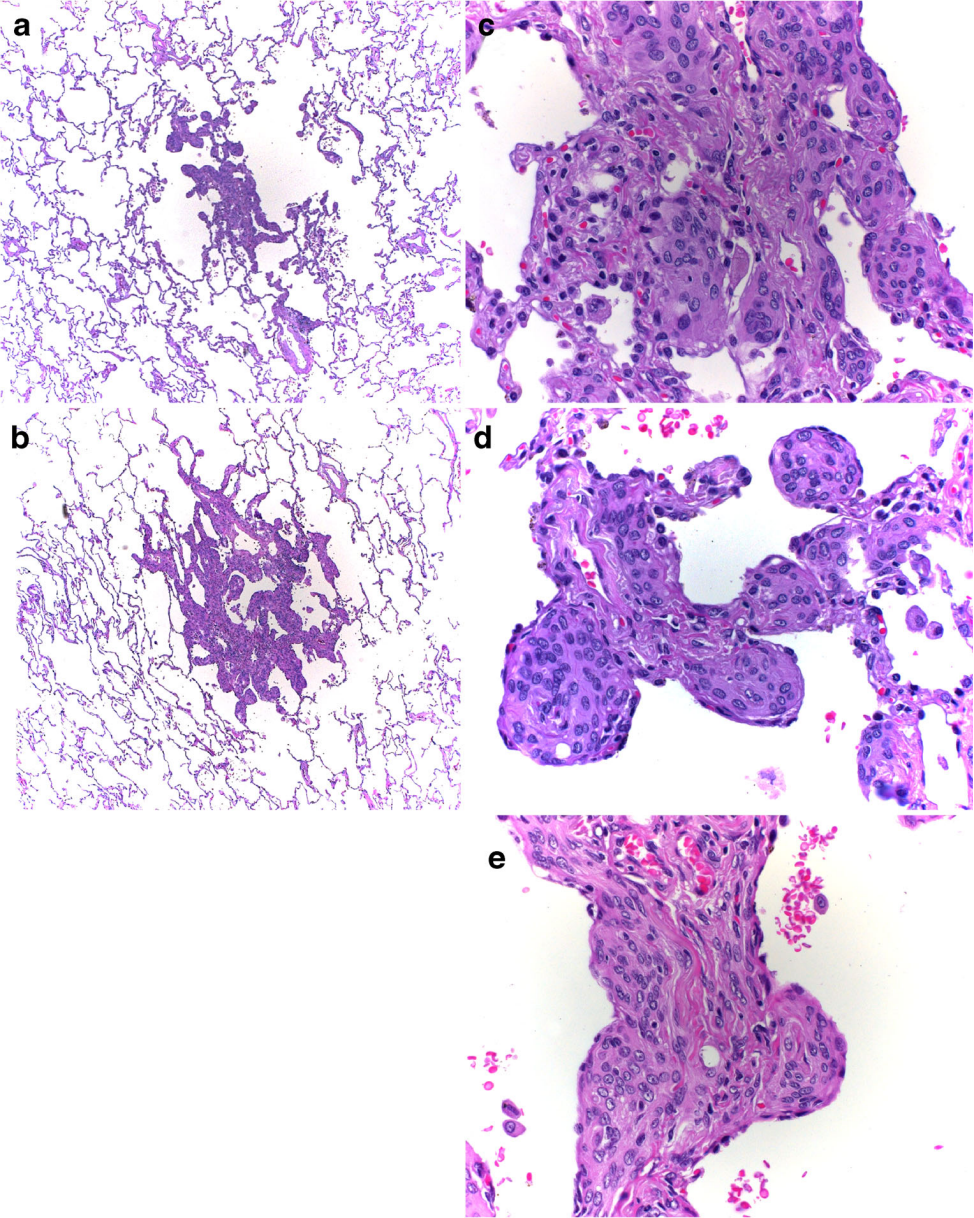
Case 2: **a** and **b** show an overview of two of the multifocal lesions. H&E, × 50. In **c**, **d**, and **e**, higher magnification from several of these lesions is shown. Note the epithelioid cells, resembling diffuse neuroendocrine hyperplasia or also multiple nodular pneumocyte hyperplasia. In **c** and **e**, the differentiation into smooth muscle cells is suggested. H&E, × 200

Besides the adenocarcinoma, only diffuse infiltrations were seen. On low magnification, these sheets of presumable epithelial or epithelioid cells resembled neuroendocrine cell or multinodular pneumocyte hyperplasia (Fig. 3a, b). On higher magnification, the cells presented with round slightly enlarged nuclei and an eosinophilic cytoplasm (Fig. 3c, d, e). Clear cells were rare. These proliferations, too, were intimately associated with blood vessels (Fig. 2e). Focally, the cells changed to a more spindle cell morphology and presented cytoplasmic filamentous structures (Fig. 2c, e).

**Fig. 4:**
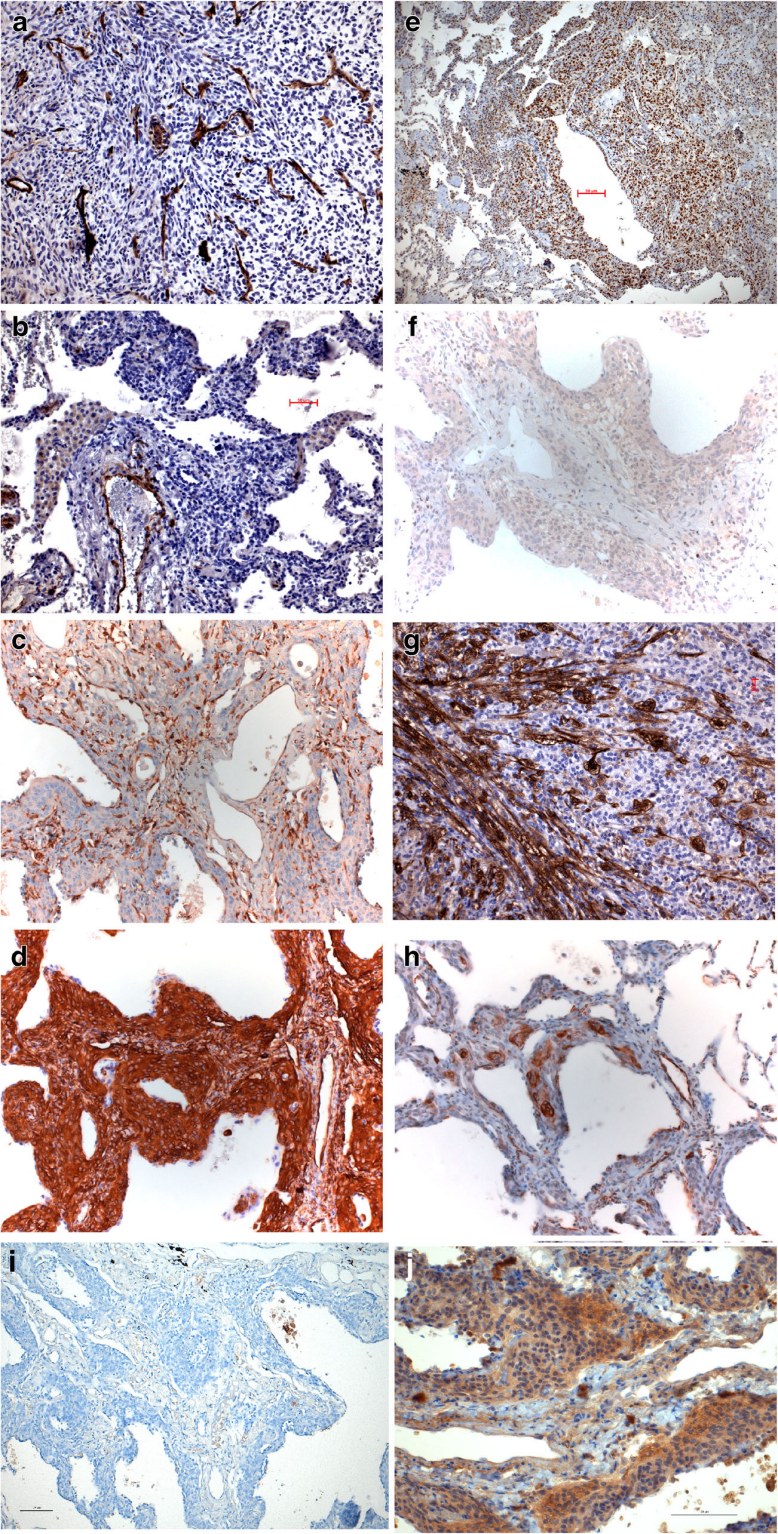
Immunohistochemistry for both cases: **a** and **b** CD31 showing the vascular network in one nodule (**a**) and the diffuse infiltration (**b**) of case 1, bar 50 μm; **c** CD31 expression in case 2, × 200. **d** Expression of vimentin in one of the lesions of case 2, × 200. Expression of VEGFR3 (**e**) in case 1 and VEGFR2 (**f**) in case 2. In VEGFR3, an antibody was used which detects the C-terminal end of the receptor; the nuclear reaction might be interpreted that the kinase domain is translocated into the nucleus, whereas the expression of VEGFR2 is membranous. Bar 50 μm and × 200. **g** Expression of SMA in the tumor of case 1 and in the proliferation of case 2 (**h**). Bar 20 μm and × 200. **i** Negative staining for cathepsin K and positive expression of PDGFRB (**j**), both in case 2. Bars 100 μm

Immunohistochemistry of both reticulonodular lesions including the tumor in case 1 (Table 2) was negative for two different pan-cytokeratin and cytokeratin 7 antibodies, neuroendocrine markers (CGA, Synaptophysin, PG9.5), S100 protein, vascular markers (CD31, CD34, factors VIIIAG and XIII, ERG) (Fig. 4a, b, c), podoplanin, common leukocyte antigen (CD45), calretinin, TTF1, p40, and desmin.

**Table 2:** Reaction of tumor cells from cases 1 and 2. Negativity for calretinin and podoplanin rules out mesothelioma, negativity for SMA and desmin rules out leiomyomatous tumors, and negativity for CD68, 45, and 35 rules out reticulum cell tumors. A negative reaction for S100 protein rules out nerve sheet tumors. *nd* not done, either because of limited tissue or because of negative staining in previous tissues; *f* focal

Antibody	Diffuse process OLB 2001	Tumor cells OLB 2001	Diffuse process biopsy 2006	Tumor cells OLB 2006
Patient 1				
Pancytokeratin	-	-	-	-
CD 56 (NCAM)	nd	nd	-	-
LCA CD45	nd	-	nd	-
S100 protein	nd	-	-	-
Vimentin	+	+	+	+
Desmin	-	-	nd	-
Smooth muscle actin	-	f+	-	f+
CD31	-	-	-	-
CD34	-	f+	-	-
CD68	nd	-	nd	-
Ki67	≥ 5%	≥ 5%	≥ 5%	≥ 5%
HMB45	-	-	-	f++
ER	nd	nd	nd	-
PG	nd	nd	nd	+
VEGF-A	++	++	+	+
VEGFR-2	-	-	nd	+
VEGFR-3	++	+++	nd	++
Endothel kinase Tie2	++	++	nd	++
cAMP kinase alpha	++	++	nd	nd
Hamartin	-	-	nd	nd
Tuberin	+	+	nd	nd
Calretinin	-	-	-	-
Podoplanin	-	-	-	-
Factor VIIIAG	Tu-endothelia+			
HHV8	-	-	-	-
CD35	-	-	-	-
Patient 2	Adenocarcinoma 2020	Nodular and trabecular process 2020		
Cytokeratin 7	+	-		
CAM5.2	+	-		
AE1/3	+	-		
Chromogranin A	-	-		
Synaptophysin	-	-		
Estrogen receptor	-	-		
Progesterone receptor	-	+		
TTF1	+	-		
P40	-	-		
HMB45	-	-		
MelanA	-	-		
MITF	-	-		
MIB1	12%	< 1%		
Vimentin	-	+		
Desmin	-	-		
Factor XIII	-	-	Vascular structures within nodules	
ERG	-	-		
S100 protein	-	-		
SMA	-	+		
Caldesmon	-	Single cells+		
MyoFD5	-	Single cells+		
VEGFR2	-	+		
VEGFR3	-	-		
Cathepsin K	-	-		
PDGFRB	-	+

Both lesions and the tumor were in part positive for smooth muscle actin (SMA) (Fig. 4g, h), progesterone receptor, and vimentin (Fig. 4d). Either vascular endothelial growth factor receptor 2 or 3 (VEGFR2, VEGFR3) (Fig. 4e, f), vascular endothelial growth factor A (VEGF-A), or endothel kinase Tie2 was positive. Tuberin was positive in the diffuse process in case 1; single cells in case 2 were stained positively for caldesmon and MyoFD5. HMB45 was positive in few tumor cells of case 1, but negative in case 2. MITF and Melan A were negative in case 2 and hamartin in case 1 (for details, see Table 2). Cathepsin K was negative in case 2, whereas platelet-derived growth factor B (PDGFRB) (Fig. 4i, j) was positive in the lesions from case 2. Case 1 could not be evaluated because the tumor tissue was completely consumed.

## Discussion

Both cases presented with a multifocal diffuse infiltration, in case 1 associated with small nodules. Four years later, a large tumor was seen on CT scan. An epithelial nature of the tumor cells was excluded by immunohistochemical stains for epithelial markers. For these lesions, several differential diagnoses were raised: A lymphoid lesion was excluded in case 1, and a neuroendocrine proliferation in case 2. Because of the intimate association with blood vessels in both cases, different endothelial markers were investigated, which highlighted the blood vessels within the proliferation, but were negative for the tumor cells. A pure smooth muscle cell proliferation was excluded, because many cells were negative for myogenic markers; however, cells within these lesions focally stained for SMA. Precursor lesions for a solitary fibrous tumor in case 1 could be excluded by negativity for CD34. Solitary fibrous tumor can occur within the lung and sometimes present with a hemangiopericytic pattern; however, additional to CD34, which was negative in both cases, they express STAT6, which was negative in case 2 also. Case 2 resembled a multifocal pneumocyte hyperplasia [23], but as it was negative for cytokeratin and TTF1 could be excluded. Diffuse neuroendocrine hyperplasia also can mimic the lesions in case 2. However, a negative reaction for neuroendocrine markers and cytokeratin helped to exclude this entity too.

Hemangioma or glomangioma patterns were not present in both cases. The majority of the diffuse proliferation in both cases was positive for the mesenchymal marker vimentin, and there were few clear cells positive for HMB45 in case 1. A PEComa could be excluded in case 2 due to negativity for MITF and cathepsin K [5, 18]. This all pointed to a pericytic differentiation. Pericytes in the fetal period are responsible for the formation of the outer vascular wall, whereas the endothelia are formed from bone marrow–derived mesenchymal precursor cells [2]. Pericyte precursor cells differentiate into smooth muscle cells, pericytes, and perivascular epithelioid cells (PEC) [3, 24]. This can be seen in both cases: focally, these cells differentiated into smooth muscle cells, and in the tumor, focal HMB45-positive perivascular epithelioid cells (PEC) are seen. In the diffuse proliferation in case 1, there were some associated endothelial proliferations with doubling of capillaries, but again no classical angiomatous pattern, such as endothelial-lined vascular spaces [1, 33], which excludes a hemangiopericytoma. Hemangiopericytomas have been described in the lung, expressing smooth muscle cell and endothelial markers [1, 4, 12, 15, 17, 29, 32, 33]. Glomus tumors and glomangiopericytoma have also been reported [9, 16, 27, 28]. Our second case somehow resembled a glomus tumor or glomangiomyoma, but it lacked the angiomatoid structures; with respect to glomangiopericytoma, the prominent thin- and thick-walled blood vessels were missing. In addition, desmin staining was absent in both cases. As no extensive fibrous tissue was noted and also only a minority of tumor cells expressed SMA, an angiocentric myofibromatosis could be excluded [7]. But both cases very nicely show the evolution of this lesion from pericytosis (diffuse process in both cases) to myopericytosis to finally myopericytoma in case 1. Myopericytoma is a rare neoplasm which can arise from soft tissues, but also within organ systems [10, 11]. A few case reports are available where the location was in the lung [6, 21, 30]. Myopericytoma is one of four entities listed within the group of pericytic tumors besides glomangiopericytoma, infantile myofibromatosis, and hemangiopericytoma [11, 13]. Our first case morphologically fulfilled the classical pattern with primitive spindle cells and focal differentiation into smooth muscle as well as perivascular epithelioid cells (PEC) within the tumor. The second case presented with uniform epithelioid cells, without PEC, but focal myogenic differentiation. The immunohistochemical profile of both tumors was in line with what has been reported for myopericytomas, namely expression of smooth muscle actin and vimentin [7, 24]. Recently an activating mutation for PDGFRB was reported for myopericytoma [13]. A low level of expression was reported. In our case 2, we found a moderate protein expression by immunohistochemistry.

Single case reports of myopericytomas have been reported in the lung. However, our cases are unique in the sense that they represent precursor lesions with a quite different morphological appearance, and in addition present with a tumor, arising out of this precursor lesion. In addition, we also present evidence for a more specific therapeutic intervention, using targeted therapy, which might inhibit angiogenesis. As both tumors are likely driven by VEGF receptors, a more selective therapy available nowadays could inhibit these receptors and interfere not only with growth signaling in the large tumor but also in the diffuse proliferation, preceding the tumor.

VEGF receptors play an important role in fetal organogenesis of the lung. VEGFR1 is predominantly driving angiogenesis in the central vascular system, whereas VEGFR2/3 plays a major role in directing the growth of the peripheral capillary and lymphatic net [14, 25]. VEGFR2/3 also plays a role in low- and high-grade angiosarcomas [19, 31]. Since pericytes and precursors in a fetal lung are also under the control of these growth factors, our findings suggest a possible way for treatment. Initially in case 1, the antibody against VEGF, bevacizumab, approved 2005, was chosen. As inhibitors of angiogenesis carry a risk for bleeding, thrombosis, and hypertension [22], a drug targeting VEGFR 2 and 3 might selectively block the receptor. Pazopanib, a TKI inhibiting VEGFR, PDGFR, and c-KIT, approved for advanced renal cell carcinoma and advanced sarcoma of soft tissue, might be an option. Ramucirumab, a monoclonal antibody selective against VEGFR2 and approved for advanced gastric cancer, could be another option. A third option might be nintedanib, a TKI targeting PDGFR, FGFR, and VEGFR in this rare kind of tumors.

Additional tissues sections from case one could be relocated and stained for MITF, cathepsin K, and PDGFRB. MITF and cathepsin K were both negative in the myopericytoma and in myopericytosis. The reaction for PDGFRB was positive in the epitheloid cells of the myopericytoma, but negative in myopericytosis.
